# Validation of an Anti-Müllerian Hormone Cutoff for Polycystic Ovarian Morphology in the Diagnosis of Polycystic Ovary Syndrome in the HARMONIA Study: Protocol for a Prospective, Noninterventional Study

**DOI:** 10.2196/48854

**Published:** 2024-02-06

**Authors:** Terhi T Piltonen, Deirdre Allegranza, Martin Hund, Katharina Buck, Johanna Sillman, Riikka K Arffman

**Affiliations:** 1 Department of Obstetrics and Gynecology Oulu University Hospital University of Oulu (MRC Oulu, Finland) Oulu Finland; 2 Research Unit of Clinical Medicine Medical Research Center, Oulu University Hospital University of Oulu (MRC Oulu, Finland) Oulu Finland; 3 Roche Diagnostics International Ltd Rotkreuz Switzerland; 4 Roche Diagnostics GmbH Penzberg Germany

**Keywords:** anti-Müllerian hormone, immunoassay, polycystic ovarian morphology, polycystic ovary syndrome, transvaginal ultrasound

## Abstract

**Background:**

Polycystic ovary syndrome (PCOS) is one of the most common endocrine disorders in women and is diagnosed using the Rotterdam criteria, including diagnosis of polycystic ovarian morphology (PCOM) by transvaginal ultrasound (TVUS). Due to high cost, availability, and the impact of the operator and ultrasound equipment on the reliability of the antral follicle count (AFC) by TVUS, an unmet need exists for a diagnostic test to determine PCOM without TVUS. A strong positive correlation between elevated anti-Müllerian hormone (AMH) levels and AFCs has been demonstrated in women with PCOS. In addition, recent updates to the international evidence-based PCOS guidelines state that serum AMH can be used as an alternative to TVUS-determined AFC, in the diagnosis of PCOM. The retrospective APHRODITE study derived and validated an AMH cutoff of 3.2 ng/mL for the Elecsys AMH Plus or Elecsys AMH assays (Roche) to diagnose PCOM in patients with PCOS.

**Objective:**

This study aims to further validate, in an independent prospective cohort, the AMH cutoff (3.2 ng/mL) for PCOM determination, which was previously derived and validated in the APHRODITE study.

**Methods:**

This large, prospective, multicenter, population-based, noninterventional study will evaluate the previously established AMH cutoff for the determination of PCOM during the diagnosis of PCOS using the Elecsys AMH Plus immunoassay in an independent population. Participants were women born between July 1985 and December 1987 in Northern Finland; the study partially links to the Northern Finland Birth Cohort 1986. We assessed the enrolled women, determined with the 2023 PCOS Guidelines, for current PCOS status and divided them by phenotype if positive. Each participant had 1 study visit to collect serum samples, record clinical data, and undergo a gynecological examination including TVUS. All data were collected by highly trained midwives or trained gynecologists. Sensitivity, specificity, and agreement measures were used to validate the previously determined cutoff in the whole population and in subpopulations based on phenotype and relevant demographic or clinical factors. The minimum target sample size was approximately 1800 women, including approximately 10% with PCOS.

**Results:**

At the time of manuscript submission, participant recruitment had concluded, and 1803 women were enrolled into the study. Data collection is complete and biostatistical analysis is planned for 2023.

**Conclusions:**

To limit variability, there were few TVUS operators and only 2 TVUS machines of the same type. Additionally, all women who were taking oral contraceptives were excluded from the primary analysis population. Selection bias was limited as this was a population-based study and participants were not seeking treatment for PCOS symptoms. Validating the AMH cutoff in a large, population-based study will provide further evidence on the utility of the Elecsys AMH Plus or Elecsys AMH assays in PCOM diagnosis as an alternative to TVUS. Measuring AMH for PCOM diagnosis could reduce delayed or missed diagnoses due to operator-dependent TVUS examinations.

**Trial Registration:**

ClinicalTrials.gov NCT05527353; http://tinyurl.com/2f3ffbdz

**International Registered Report Identifier (IRRID):**

DERR1-10.2196/48854

## Introduction

**Figure 1:**
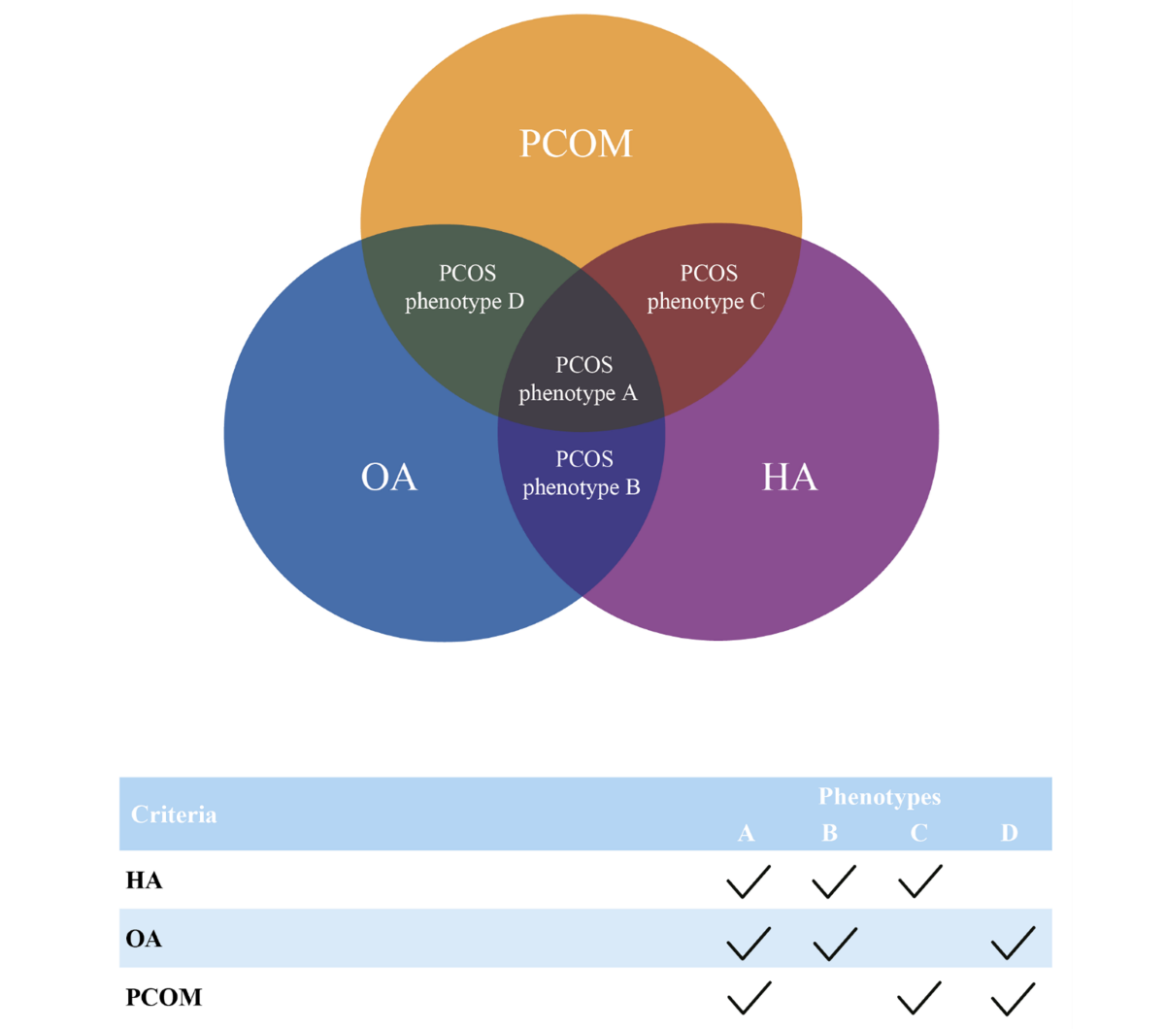
Diagnostic criteria and phenotypes of PCOS based on the Rotterdam criteria. HA: hyperandrogenism; OA: oligoanovulation or anovulation; PCOM: polycystic ovarian morphology; PCOS: polycystic ovary syndrome.

Polycystic ovary syndrome (PCOS) is one of the most common endocrine disorders affecting women and is characterized by symptoms of ovarian dysfunction and an excess of androgen, without other specific diagnoses [1-3]. PCOS is the primary cause of female anovulatory infertility and has metabolic, cardiovascular, and psychological implications [4-7]. The diagnosis of PCOS is based on the Rotterdam criteria [8,9], where 2 or more of the following conditions should be met: oligoanovulation or anovulation (OA), clinical or biochemical signs of hyperandrogenism (HA), a combination of both, and polycystic ovarian morphology (PCOM) based on transvaginal ultrasound [10] (TVUS; Figure 1).

According to the updated guidelines [9], the diagnosis of PCOM is based on a TVUS with a finding of ≥20 follicles of 2-9 mm in size in at least 1 ovary or increased ovarian volume ≥10 mL, but determination of the antral follicle count (AFC) by TVUS depends on the operator and ultrasound equipment used [11-17]. Due to the expense of the equipment and the regularity of updates needed, in addition to the very high level of specialized training required by operators, TVUS is not available for many physicians seeing women with symptoms of PCOS (eg, general practitioners, gynecologists, and endocrinologists). Thus, there is significant underdiagnosis of PCOS [18] along with a substantial delay in receiving a diagnosis [19-22]; therefore, an unmet medical need exists for a diagnostic test to determine PCOM without the need for TVUS.

Anti-Müllerian hormone (AMH) is a regulator of follicle recruitment from the primordial follicle pool and inhibitor of follicular growth that is expressed by granulosa cells in the preantral and small antral follicles [23,24]. Serum AMH levels correlate well with the 2-9 mm antral follicles found in TVUS examination in the diagnosis of PCOM [25,26]. Elevated levels of AMH are observed in women with PCOS [27-30], and a strong correlation between elevated levels of AMH and increased AFCs has been demonstrated [12,14,26,29], supporting the use of AMH as a biomarker for PCOM.

In the recent, retrospective, case-control APHRODITE study, a cutoff of 3.2 ng/mL for the Elecsys AMH Plus or Elecsys AMH assays (Roche Diagnostics International Ltd) was derived and validated to identify PCOM as part of PCOS diagnosis in women aged 25-45 years [28]. Moreover, a recent study reported the usability of the Elecsys AMH assay to identify PCOS cases in large epidemiological data sets [31]. All previous findings support the use of AMH measurement using the Elecsys AMH Plus assay as a substitute for AFC in the determination of PCOM, thereby reducing the need for TVUS procedures [28,32].

Since APHRODITE was a retrospective, case-control study that validated the derived cutoff in a population with an increased risk of PCOS, it is of interest to further validate the derived cutoff in a prospective, independent, population-based cohort. By validating AMH levels in a large, population-based study using the Roche Elecsys AMH Plus assay, we aim to provide clinicians with the ability to identify PCOM as part of a PCOS diagnosis using a simple blood test, thereby making diagnosis of the disorder more accessible in a primary care setting. Although it is not anticipated that TVUS as a diagnostic method for PCOM will be discarded from PCOS guidelines, AMH testing could be adopted as an alternative method, particularly in primary care.

## Methods

### Study Design

The prospective, multicenter, population-based, noninterventional HARMONIA (Human Anti-Müllerian Hormone for Diagnosis of PCOS) study aims to validate the AMH cutoff determined and validated in the APHRODITE study for the determination of PCOM during the diagnosis of PCOS using the Elecsys AMH Plus immunoassay. Study enrollment was conducted between May 2020 and October 2022 at 2 sites in Finland: Oulu University Hospital, Department of Obstetrics and Gynecology, Oulu, and Helsinki University Hospital, Helsinki.

### Participants

The target population was women born in Northern Finland between July 1, 1985, and December 31, 1987; the study partially links to the Northern Finland Birth Cohort (NFBC) 1986 study population. The prevalence of PCOS in this population is expected to be approximately 10% when applying the diagnostic criteria for PCOS outlined in the 2018 international PCOS guideline [9,33]. The COVID-19 pandemic resulted in a lower enrollment rate than originally expected, and so the cohort was expanded to include a random cohort of women born in the same geographical area up to 18 months after those in the NFBC 1986, to ensure enrollment met the minimum target sample size of approximately 1800 individuals. As the study is population-based, all nonpregnant women from the target population were invited to participate in the study.

Participants were excluded if they were unwilling to undergo gynecological examination including TVUS, refused to have blood drawn, or did not consent to sharing their personal data with Roche Diagnostics International Ltd, who completed the AMH measurement and analysis. Additionally, women who were taking oral contraceptives at the time of study commencement will be excluded from the primary analysis population.

PCOS cases were defined as women fulfilling 2 or more Rotterdam criteria [8,9]. For each participant, the current PCOS status was assessed. PCOS positive cases were further divided into the following phenotypes: phenotype A (HA+, OA+, PCOM+), phenotype B (HA+, OA+, PCOM-), phenotype C (HA+, OA-, PCOM+), and phenotype D (HA-, OA+, PCOM+; Figure 1). PCOM in PCOS cases included the PCOS A, C, and D phenotypes. PCOS phenotype B cases by definition did not meet the criteria for PCOM based on TVUS (phenotype B accounted for approximately 3% of PCOS cases in the APHRODITE study) and were not included in the case group for the primary objective. Controls were defined as women with negative PCOM with an AFC <20, an ovarian volume <10 mL, and no other diagnostic features of PCOS according to the Rotterdam criteria.

Each participant had 1 study visit during which serum samples were collected through blood draw (taken at any stage of the menstrual cycle), clinical data (including questionnaire responses) were recorded, and a gynecological examination including TVUS (Ultrasound System HS60, Samsung Healthcare) was performed to determine PCOM status; all assessments were evaluated by a clinician (Table 1) [34]. All data were collected by highly trained midwives or gynecologists.

**Table 1:** Baseline characteristics to be recorded by questionnaire and clinical assessment.

Baseline characteristic	Questionnaire	Clinical assessment
General information and medical history	Participant identification and date of consent Age at the study visit Race Education History of radiation or chemotherapy (yes or no) or treatment Long-term illnesses diagnosed by a doctor (self-reported) Bothersome hair loss experience Acne (former or current)	Confirmation of fasting for 12 hours before the clinic visit
Medication use	Medication use (current) Use and type of hormonal contraceptives (oral, patch, or intrauterine device; former or current)	N/A^a^
Intoxications	Smoking (current, former, or never; number of cigarettes) Alcohol (units per week)	N/A
Obstetric history	Menarche Cycle (regular, irregular, or absent) Shortest and longest menstrual cycle length (days) Last menstrual cycle Number of live births, fetus mortus, miscarriages, extrauterine pregnancy, and pregnancy terminations Fertility treatment Breastfeeding status (no, exclusively, or partially) Delivery within 6 months from the study visit	From the transvaginal ultrasound report: Endometrial thickness Visualization status of the ovaries Ovarian length, width, height, and volume Antral follicle count Dominant follicle presence Presence of corpus luteum and/or cysts >25 mm and/or other possible reason for increase of the ovarian volume
Anthropomorphic	N/A	Height Weight Waist circumference Hip circumference Systolic blood pressure or diastolic blood pressure Gynecological ultrasonography (polycystic ovarian morphology) Hirsutism (Ferriman-Gallwey-Score) Acne severity grading (1-5) per the Global Acne Severity Scale [34]. Images of the participant will be assessed by a dermatologist

^a^N/A: not applicable.

### Sample Processing

A total volume of approximately 5 mL of serum was collected using VACUETTE Tube 9 mL CAT serum clot activator tubes (Greiner Bio One) and stored at –80 °C. Aliquots were sent to the Nordlab in Oulu for measurement of testosterone by liquid chromatography-mass spectrometry (Sciex Qtrap 5500, Ab Sciex). Additional measurements were performed on-site at Oulu (testosterone, sex hormone-binding globulin, follicle-stimulating hormone, luteinizing hormone, thyroid-stimulating hormone, and prolactin) on the COBAS e 411 analyzer (Roche Diagnostics International Ltd), which was used to assess some of the PCOS criteria. Additional aliquots were shipped to Roche Diagnostics GmbH, Penzberg, Germany, for determination of AMH biomarker levels (Textbox 1). AMH levels were measured using the Elecsys AMH Plus assay on the COBAS pro e 801 analyzer (Roche Diagnostics International Ltd). The measuring range of the Elecsys AMH Plus assay is 0.01-23 ng/mL and the coefficient of variance for repeatability is less than 3% [35]. Participants with AMH levels more than 3.2 ng/mL (the cutoff determined and validated in the APHRODITE study [28]) were classified as AMH PCOM positive, and those with results ≤3.2 ng/mL were classified as AMH PCOM negative.

**Laboratory parameters to be measured from blood samples.**

**Parameters to be measured**

Hemoglobin A1c
Complete blood count
Ferritin
Fasting plasma glucose
Fasting total cholesterol
Fasting plasma high-density lipoprotein
Fasting plasma low-density lipoprotein
Fasting plasma triglycerides
Plasma alkaline phosphatase
Plasma albumin
Fasting serum C-peptide
Plasma alanine aminotransferase
Plasma amylase
Plasma aspartate aminotransferase
Plasma bilirubin
Plasma gamma glutamyltransferase
Plasma creatinine
Plasma uric acid
Fasting serum insulin
Serum high-sensitivity C-reactive protein
Testosterone
Sex hormone-binding globulin
Follicle-stimulating hormone
Luteinizing hormone
Thyroid-stimulating hormone
Prolactin
Anti-Müllerian hormone
Dehydroepiandrosterone sulfate

### Data Analysis

At the time of publication submission, the trial was still underway, although data collection was complete. During the planned biostatistical analysis, baseline characteristics and biomarker data will be analyzed for all participants, and by PCOS phenotype and case or control groups. Additionally, the baseline characteristics will be compared between cases and controls using statistical tests, such as Mann-Whitney *U* tests or chi-square tests.

The primary objective of this study, conducted in the PCOM positive population, will be validating the AMH cutoff for PCOM determined in the APHRODITE study. Agreement measures will be calculated, and tables produced to estimate the performance (ie, sensitivity, specificity, and receiver operating characteristic curve) of the prespecified Elecsys AMH cutoff for the prediction of PCOM status. In addition, performance estimates (sensitivity, specificity, and agreement tables) of the Elecsys AMH Plus cutoff will be performed within subpopulations (phenotype and potentially relevant demographic or clinical factors).

### Sample Size and Power

The total sample size for the primary analysis was a minimum of approximately 1800 women, of whom approximately 10% will be PCOS positive cases; however, some individuals may need to be excluded from the primary objective analysis due to hormonal contraceptive use. As PCOS phenotype B cases do not meet the criteria for PCOM based on TVUS, some PCOS cases will also be excluded. In addition, some women may need to be excluded if their PCOS or PCOM status cannot be determined (eg, due to the inability to adequately visualize the ovaries). Assuming a significance level of 0.05 (1-sided lower CIs) and a joint power of 80%, approximately 55-88 PCOS positive cases and approximately 164-262 PCOS negative cases are needed to achieve agreements of 65% and 70%, if the true percentages of agreement are 79%-82.5% and 78%-80%, respectively.

### Ethical Considerations

The study complies with all relevant national regulations and institutional policies and was performed in accordance with the principles of the Declaration of Helsinki. Ethical approval was provided by the Ethical Committee of the Northern Ostrobothnia Hospital District (EETTMK 47/2019), and all participants were required to give informed consent for the use of their collected data for scientific purposes. The trial was registered (NCT05527353) on September 2, 2022.

## Results

The first patient provided consent on May 7, 2021, and the last patient provided consent on October 28, 2022; therefore, participant recruitment has been completed. At the time of manuscript submission, 1803 women had been enrolled into the study. Biostatistical analysis will commence later in 2023 and it is expected that the results will be published shortly thereafter.

## Discussion

This is the first large, prospective, population-based study to validate an AMH level cutoff for determining PCOM status in the diagnosis of PCOS.

### Strengths and Limitations

One strength of this study is that all data were collected by highly trained midwives and specialized gynecologists. The TVUS data is robust due to the limited number of TVUS experienced operators performing the ultrasound assessments, and the use of only 2 TVUS machines of the same type. In addition, while most studies on AMH testing are retrospective, using small, non–population-based cohorts from PCOS clinics, HARMONIA is a large, prospective, population-based study. Furthermore, there will be limited bias, as the study is population-based, and participants were not seeking treatment for PCOS symptoms. However, the study also has some limitations. The age of the participants is limited by the study’s linkage to the NFBC 1986 (all participants are within a 3-year age range). In the APHRODITE study, it was also found that AMH levels decreased with age among PCOS cases and controls [28]; findings from this study should therefore be evaluated in the context of these results. Furthermore, due to the geographical location of the study and the fertile age of the participants, most women will be White (with a small minority, estimated below 3%, of indigenous Sámi people), and some will be taking hormonal contraceptives. It has also previously been shown that there is some biological variability in AMH during the menstrual cycle [36]. However, a longitudinal study conducted in a population-based cohort demonstrated that AMH may be used as a surrogate marker for identification of PCOM [37]. The samples in this study will be taken at any stage of the menstrual cycle; however, we anticipate that the small variation in cycle phases will not affect the diagnostic performance of the Elecsys AMH Plus assay. Furthermore, studies have shown that women with PCOS may have a higher body mass index, which could affect the precision of TVUS results [38,39]. In addition, the global COVID-19 pandemic may have caused selection bias during enrollment in the original NFBC 1986 population, although this factor applies to the entire cohort. The study results will be analyzed and interpreted in this context.

### Benefits of the Study

By validating AMH levels in a large, population-based study, clinicians will be able to identify PCOM as part of PCOS diagnosis using a simple blood test. Thus, if TVUS is necessary only as a means to identify PCOM (rather than for other clinical reasons), this procedure can be replaced, thereby making the diagnosis of PCOM more accessible in a primary care setting. This would lead to much faster diagnoses for patients, as in some health care settings there would be no need to wait for referral, and only patients needing treatment for other specialized health care (eg, fertility, topical skin therapy, or psychological distress) would require referral to a specialist. This would also allow the common health impairments of the women affected to be viewed more holistically, rather than categorizing these women into an infertile population without further consideration of other adverse outcomes (eg, risk of developing type 2 diabetes mellitus or psychological distress). Testing AMH levels could also contribute to reducing missed diagnoses due to operator-dependent TVUS examinations. Following the validation of AMH levels in this study, we do not anticipate that PCOS guidelines will discard TVUS as a diagnostic method for PCOM, but rather advise that AMH testing be adopted as an alternative method.

## Abbreviations

AFC: antral follicle count
AMH: anti-Müllerian hormone
HA: hyperandrogenism
HARMONIA: Human Anti-Müllerian Hormone for Diagnosis of PCOS
NFBC: Northern Finland Birth Cohort
OA: oligoanovulation or anovulation
PCOM: polycystic ovarian morphology
PCOS: polycystic ovary syndrome
TVUS: transvaginal ultrasound

## Data Availability

The data sets that will be generated or analyzed during this study will be available for academic research collaborations through the research group at the University of Oulu and the corresponding author on reasonable request. The Women’s health study (WENDY) data can also be viewed [40].
